# Consumption of Dietary Fiber with Different Physicochemical Properties during Late Pregnancy Alters the Gut Microbiota and Relieves Constipation in Sow Model

**DOI:** 10.3390/nu14122511

**Published:** 2022-06-16

**Authors:** Dongdong Lu, Yu Pi, Hao Ye, Yujun Wu, Yu Bai, Shuai Lian, Dandan Han, Dongjiao Ni, Xinhua Zou, Jinbiao Zhao, Shuai Zhang, Bas Kemp, Nicoline Soede, Junjun Wang

**Affiliations:** 1 State Key Laboratory of Animal Nutrition, College of Animal Science and Technology, China Agricultural University, Beijing 100193, China; dongd.lu@hotmail.com (D.L.); yzupiyu@163.com (Y.P.); yujun@cau.edu.cn (Y.W.); yubaijlucau@163.com (Y.B.); lianlianshuai@163.com (S.L.); handandan@cau.edu.cn (D.H.); jinbiaozhao@cau.edu.cn (J.Z.); zhangshuai16@cau.edu.cn (S.Z.); 2 Key Laboratory of Biological Feed, Ministry of Agriculture and Rural Affairs, Boen Biotechnology Co., Ltd., Ganzhou 341000, China; nidj@boencorp.com (D.N.); jeff@bo-en.com (X.Z.); 3 Adaptation Physiology Group, Department of Animal Sciences, Wageningen University, 6700 AH Wageningen, The Netherlands; hao.ye@wur.nl (H.Y.); bas.kemp@wur.nl (B.K.); nicoline.soede@wur.nl (N.S.)

**Keywords:** defecation frequency, dietary fiber, different physicochemical properties, late pregnancy, intestinal motility, *Turicibacter*

## Abstract

Constipation is a common problem in sows and women during late pregnancy. Dietary fiber has potential in the regulation of intestinal microbiota, thereby promoting intestinal motility and reducing constipation. However, the effects of fibers with different physicochemical properties on intestinal microbe and constipation during late pregnancy have not been fully explored. In this study, a total of 80 sows were randomly allocated to control and one of three dietary fiber treatments from day 85 of gestation to delivery: LIG (lignocellulose), PRS (resistant starch), and KON (konjaku flour). Results showed that the defecation frequency and fecal consistency scores were highest in PRS. PRS and KON significantly increased the level of gut motility regulatory factors, 5-hydroxytryptamine (5-HT), motilin (MTL), and acetylcholinesterase (AChE) in serum. Moreover, PRS and KON promoted the IL-10 level and reduced the TNF-α level in serum. Furthermore, maternal PRS and KON supplementation significantly reduced the number of stillborn piglets. Microbial sequencing analysis showed that PRS and KON increased short-chain fatty acids (SCFAs)-producing genera *Bacteroides* and *Parabacteroides* and decreased the abundance of endotoxin-producing bacteria *Desulfovibrio* and *Oscillibacter* in feces. Moreover, the relative abundance of *Turicibacter* and the fecal butyrate concentration in PRS were the highest. Correlation analysis further revealed that the defecation frequency and serum 5-HT were positively correlated with *Turicibacter* and butyrate. In conclusion, PRS is the best fiber source for promoting gut motility, which was associated with increased levels of 5-HT under specific bacteria *Turicibacter* and butyrate stimulation, thereby relieving constipation. Our findings provide a reference for dietary fiber selection to improve intestinal motility in late pregnant mothers.

## 1. Introduction

Constipation of sows during pregnancy is a common problem affecting the reproductive performance of sows in commercial pig farms [1,2]. About 64.6% of sows had moderate to severe constipation according to an investigation of gestating sows [3]. Constipation can not only cause pain and discomfort in periparturient sows and prolong the farrowing duration but also increase the absorption of bacterial endotoxin in the intestinal tract, leading to postpartum galactosis syndrome [4]. Furthermore, constipation-induced dysbiosis of the intestine can exacerbate oxidative stress and inflammatory responses during parturition [1,5]. Constipation may be caused by unhealthy gastrointestinal metabolism and a disordered intestinal nervous system [6]. Moreover, the diet structure, use of movement-limiting stalls, restricted feeding, and large volume of the uterus at the end of gestation increase the chance of constipation in late pregnant sows [4,7]. Therefore, a proper strategy that can relieve constipation in late-gestation sows is needed. 

The gastrointestinal regulation-related peptides and gut neurotransmitters are considered as crucial regulatory hormones in colonic hypomotility during pregnancy [8,9]. Recently, some studies revealed that the composition of the intestinal microbiome influences colonic transit and fecal consistency [10,11], since specific microbial genera or families can stimulate the release of gut motility regulatory hormones [12]. Furthermore, strong correlations between defecation patterns (stool consistency and frequency) and the fecal microbiota composition have been found in constipated humans and loperamide-induced constipated mice [11,13]. Therefore, the regulation of intestinal microbiota may be an effective way to relieve the constipation of sows at the end of gestation. 

Dietary fibers are actively involved in the regulation of the gut microbiome. Studies in pigs and humans have found positive effects of dietary fibers on the relief of constipation, which was accompanied by changes in the microbiome [14,15]. Different sources of fiber have different physicochemical properties [16] and differences in the physical and fermentative characteristics of fibers influence their potential to regulate the intestinal microbiome [17,18,19]. It is, therefore, important to study the effects of fiber sources with different properties on constipation and their microbial-regulating function. Three different sources of fibers were screened in our laboratory: konjaku flour (KON), mainly consisting of glucomannan, which is polymerized by the β-1,4 glycosidic bond, and resistant starch (PRS), which mainly consists of type 3 resistant starch, obtained by gelatinization followed by recrystallization of amylose and amylopectin [20,21,22]. Lignocellulose (LIG) mainly consists of cellulose, which can be slowly fermented in the hindgut, but it can still promote the abundance of some beneficial microbes [18,23]. PRS showed higher concentrations of formic acid and lactate than LIG and KON, whereas KON showed higher concentrations of propionate and butyrate than LIG and PRS in in vitro fermentation experiments [18]. Moreover, the in vitro fermentation results showed that the *Anaerovibrio* and *Erysipelatoclostridium* abundances were higher in KON, whereas the *Proteiniclasticum* abundance was higher in PRS [18]. Although this in vitro study showed that the three different sources of fibers differentially regulate the microbiome, the effects on the intestinal microbiome and constipation in sows during late gestation are unknown.

In this study, three fiber sources were selected based on their various physicochemical properties to investigate their effects on the constipation of sows (defecation frequency, fecal consistency, and fecal moisture) during late gestation, and their microbial-mediated mechanisms, with the purpose of providing a reference for dietary fiber selection for sows during late gestation.

## 2. Materials and Methods

This study followed the Laboratory Animal Welfare and Animal Experimental Ethical Inspection Committee in China Agricultural University (AW51211202-1-1). 

### 2.1. Animals and Experimental Designs

A total of 80 multiparous sows (Yorkshire × Landrace; ages ranged from 3–6 parity) were used in this study. Sows were weighed on day 84 of gestation and allotted to 4 treatments based on their body weight and parity in a complete block design. From day 85 of gestation till farrowing, sows were either fed a control gestation diet (CON, a corn-soybean meal basal control diet), or one of 3 diets in which wheat bran was replaced by lignocellulose (1.5%, LIG), resistant starch diet (2%, PRS), or 2% konjaku flour (2%, KON). The total dietary fiber (TDF) fraction was the same in all groups and all diets were formulated to meet the nutrient requirements of NRC (2012) for sows (Table 1). During the experimental period, sows were housed in individual gestation stalls, and fed three times a day (at 7:00, 11:00, and 17:30). Sows in CON, LIG, PRS, and KON received 3.00, 3.03, 3.04, and 3.04 kg/day of diets, respectively, to achieve the same digestible energy (DE) intake. On day 107 of gestation, sows were moved to the farrowing rooms, where they were housed in individual farrowing crates. All sows had free access to water through the whole experimental period. 

### 2.2. Monitoring of the Defection Frequency, Fecal Consistency Score, and Fecal Moisture during Late Pregnancy

The defecation frequency of sows was recorded from day 109 to day 114 of gestation, and the defecating times per day were assessed between 7:00 A.M. to 7:00 A.M. each day. From the morning of day 109 to day 114 of gestation, the fecal score after defecation was ranked using the visual qualitative evaluation method described by the Bristol Stool Scale (BSS) [24] and adjusted by Tan et al. for use in sows [14]: 0 = absence of feces, 1 = dry and pellet-shaped, 2 = between dry and normal, 3 = normal and soft but firm and well-formed, 4 = between normal and wet, still formed but not firm, and 5 = very wet feces, unformed and liquid. Subsequently, fresh feces were collected after defecation from each sow from day 109 to day 114 of gestation and immediately transported to the lab for moisture detection. The measurement of fecal moisture was executed according to Shang et al. [17]. In brief, approximately 200 g of fecal samples was oven-dried at 103 °C for 72 h. The sample weights before and after oven drying were recorded.

### 2.3. Blood and Fecal Sample Collection

Systemic blood samples were collected from 7 randomly selected sows in each group from the ear vein before morning feeding at day 112 of gestation. Blood samples were collected in 10 mL tubes filled with heparin and centrifuged at 3000× *g*, 4 °C for 10 min to obtain the serum, and then the serum samples were stored at −80 °C before analysis. Immediately following the blood sample, the same 7 sows from each group were used for the fecal sample. By massaging the rectum, about 10 g of fecal sample from each sow was collected and immediately frozen in liquid nitrogen, and then stored at −80 °C within 72 h after collection until DNA extraction and SCFAs measurement.

### 2.4. Reproductive Performance and Parturition Duration Recording 

During farrowing, the parturition duration of sows was recorded, which is the interval between the birth of the first piglet and the birth of the last piglet. The number of piglets born in total, born alive, and stillborn, and the individual birth weights were recorded after farrowing.

### 2.5. Fecal DNA Extraction, 16S rRNA Amplification, and Sequencing

Microbial DNA in the fecal samples was extracted using the QIAamp Fast DNA Stool Mini Kit (Qiagen, Tübingen, Germany) according to the manufacturer’s protocol. The DNA extract was checked on 1% agarose gel, and the DNA concentration and purity were determined with a NanoDrop 2000 UV-vis spectrophotometer (Thermo Scientific, Wilmington, DE, USA). Microbial community genomic DNA was extracted from 21 samples using the E.Z.N.A. soil DNA Kit (Omega Bio-Tek, Norcross, GA, USA) according to the manufacturer’s instructions. The V3-V4 region of the 16S rRNA gene was amplified with a universal primer of 338F (5′-ACTCCTACGGGAGGCAGCAG-3′) and 806R (5-GGACTACHVGGGTWTCTAAT-3′) by an ABI GeneAmp 9700 PCR thermocycler (ABI, Foster City, CA, USA). The PCR amplification of the 16S rRNA gene was performed as follows: initial denaturation at 95 °C for 3 min, followed by 27 cycles of denaturing at 95 °C for 30 s, annealing at 55 °C for 30 s and extension at 72 °C for 45 s, and single extension at 72 °C for 10 min, with the end at 4 °C. The PCR mixtures contained 5 × TransStart Fas tPfu buffer 4 μL, 2.5 mM dNTPs 2 μL, forward primer (5 μM) 0.8 μL, reverse primer (5 μM) 0.8 μL, TransStart FastPfu DNA Polymerase 0.4 μL, template DNA 10 ng, and finally ddH2O up to 20 μL. The PCR product was extracted from 1% agarose gel and purified using the AxyPrep DNA Gel Extraction Kit (Axygen Biosciences, Union City, CA, USA) according to the manufacturer’s instructions and quantified using a Quantus™ Fluorometer (Promega, Madison, WI, USA).

### 2.6. Analysis of 16S rRNA Sequencing Data 

Purified amplicons were pooled in equimolar, and paired-end sequenced on an Illumina MiSeq PE300 platform (Illumina, San Diego, CA, USA) according to the standard protocols of Majorbio Bio-Pharm Technology Co. Ltd. (Shanghai, China). The raw 16S rRNA gene sequencing reads were demultiplexed, quality-filtered using Fastp (version 0.20.0, https://github.com/OpenGene/fastp, accessed on 24 May 2021), and merged using FLASH version 1.2.7 (Adobe, San Jose, CA, USA). Then, the high-quality sequences were de-noised using the DADA2 plugin in the Qiime2 (version 2020.2, https://qiime2.org, accessed on 24 May 2021) pipeline with recommended parameters, which obtained a single-nucleotide resolution based on the error profiles within samples. Taxonomy was assigned to each amplicon sequence variant (ASV) generated by the naive Bayes consensus taxonomy classifier implemented in Qiime2 and the SILVA 16S rRNA database (v138, http://www.arb-silva.de, accessed on 24 May 2021). Analyses of the 16S rRNA microbiome sequencing data were performed using the online platform of Majorbio Cloud Platform (cloud.majorbio.com, accessed on 24 May 2021). 

### 2.7. Determination of the SCFAs Profile in Feces 

Determination of the SCFAs in the feces was performed according to the ion chromatography method described by Wu et al. [25]. Briefly, 0.1 g of feces was mixed with 8 mL of cooled, sterile deionized water, and then homogenized by magnetic rotation for 10 min. After this, the sample was centrifuged at 5000× rpm/min (4 °C) for 20 min. Then, the supernatants were diluted at 1:50, filtered through a 0.22-μm membrane, and subjected to an ion chromatography system (DIONEX ICS-3000, Thermo Fisher Scientific, Waltham, MA, USA) for SCFAs measurement.

### 2.8. Determination of Gut Motility Regulatory Factors, Endotoxin, and Inflammatory Factors in Serum 

The concentration of serum serotonin (5-HT), motilin (MTL), endothelin-1 (ET-1), acetylcholinesterase (AChE), and inflammation factors IL-6 (interleukin-6), IL-10 (interleukin-10), TNF-α (tumor necrosis factor-α), and endotoxin in serum were measured by the enzyme-linked immunosorbent assay (ELISA) kit method following the manufacturer’s instructions (Nanjing Jiancheng Bioengineering Institute, Nanjing, China). Furthermore, Nitrogen monoxide (NO) in serum was detected using a nitric oxide assay kit obtained from Nanjing Jiancheng Bioengineering Institute.

### 2.9. Statistical Analysis

The difference in the defecation parameters (fecal consistency scores, fecal moisture, and defecation frequency), reproductive parameters (stillbirth and parturition duration), SCFAs in the feces, serum concentrations of gut regulator factors (5-HT, MTL, ET-1, and AChE), serum concentrations of inflammation factors (IL-6, IL-10, and INF-α), and serum endotoxin among the four treatment groups were analyzed using one-way ANOVA and followed by Tukey’s multiple comparison test using the SPSS 20.0 software (SPSS Inc., Chicago, IL, USA). The Kruskal–Wallis test was applied for the analysis of the microbial differences between different groups at the family and genera levels. Correlations between differentiated bacteria and SCFAs and serum parameters were assessed using Spearman’s correlation test and Graphpad Prism version 5.00 (Graphpad Software, San Diego, CA, USA). Statistically significant differences are shown with asterisks as follows: * *p* < 0.05; ** *p* < 0.01; and *** *p* < 0.001.

## 3. Results

### 3.1. Effects of Different Fiber Sources on the Defecation Frequency and Fecal Parameters of Sows

**Figure 1:**
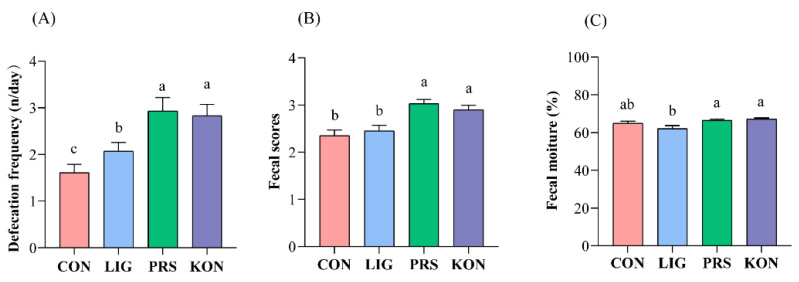
Effects of different fiber sources on the defecation frequency (**A**), fecal consistency score (**B**), and fecal moisture of sows (**C**) (*n* = 20). CON, control; LIG, lignocellulose; PRS, resistant starch; KON, konjaku flour. Differences in the superscript letters for the peer data indicate that the difference is significant (*p* < 0.05).

To explore the effects of dietary fibers on constipation in sow, the parameters: defecation frequency, stool consistency, and fecal moisture, were observed during late gestation. As shown in Figure 1A, the defecation frequency within a 24-h period was significantly higher in PRS and KON compared to CON and LIG (*p* < 0.05) while there was no difference between PRS and KON. The fecal consistency score was also affected by the treatments (Figure 1B), where PRS had the highest fecal consistency scores (*p* < 0.05). Both KON and PRS had the highest fecal moisture on day 112 of gestation (Figure 1C). The fecal water content in LIG was the lowest among the four groups (*p* < 0.05).

### 3.2. Effects of Different Fiber Sources on the Serum Gut Motility Regulatory Factors of Sows

**Figure 2:**
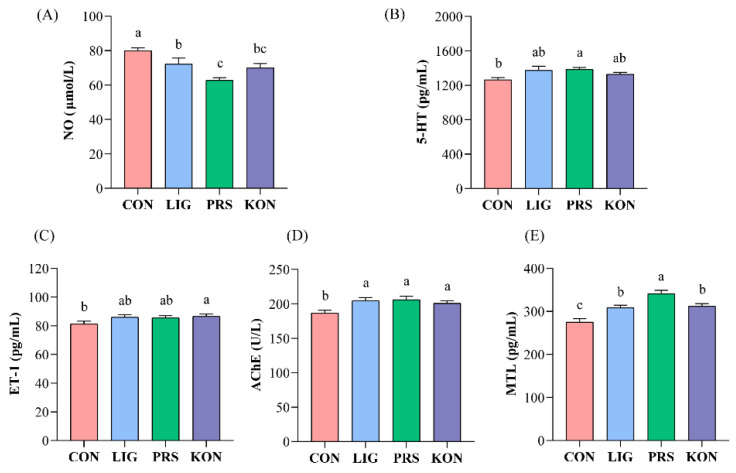
Effects of different fiber sources on the levels of 5-HT (**A**), NO (**B**), ET-1 (**C**), AChE (**D**), and MTL (**E**) in the serum of sows (*n* = 7). CON, control; LIG, lignocellulose; PRS, resistant starch; KON, konjaku flour. 5-HT, 5-hydroxytryptamine; NO, nitrogen monoxide; ET-1, endothelin-1; AChE, acetylcholinesterase; MTL, motilin. Differences between the superscript letters for the peer data indicate that the difference is significant (*p* < 0.05).

To investigate possible changes in gut motility, the gut motility regulatory hormones in serum were measured. The levels of two gut neurotransmitters: 5-HT and NO, in serum were analyzed (Figure 2A,B). The CON sows had the highest serum level of NO and the lowest level of 5-HT among the four groups. Conversely, PRS sows had the lowest level of NO and the highest level of 5-HT compared to the other groups. The levels of three gastrointestinal regulatory peptides are shown in Figure 2C–E. CON sows showed the lowest levels of MTL, AChE, and ET-1 among the four groups, and PRS sows had the highest level of AChE, MTL, and 5-HT.

### 3.3. Effects of Different Fiber Sources on the Reproductive Parameters and Parturition Duration of Sows

As shown in Table 2, the total number of born and born alive piglets, total litter weight, and average piglet birth weight were similar among the four treatments. However, KON and PRS sows tended to have a lower number of stillborn piglets (*p* = 0.08) and a significantly lower stillborn rate (*p* = 0.02) than CON sows. Moreover, the parturition duration of PRS and KON sows was significantly shorter than CON sows (*p* = 0.03).

### 3.4. Effects of Different Fiber Sources on the Composition and Diversity of the Fecal Microbiota of Sows

**Figure 3:**
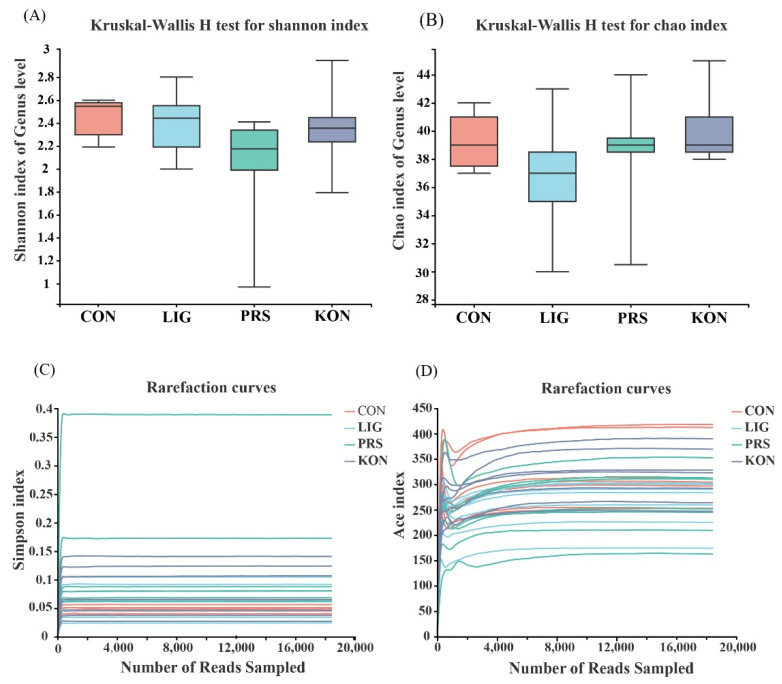
Microbial diversity in sow feces is affected by dietary fiber sources (*n* = 7). Shannon (**A**), Chao (**B**), Simpson (**C**), and Ace (**D**) indicies. CON, control; LIG, lignocellulose; PRS, resistant starch; KON, konjaku flour.

To explore the effects of the dietary fiber composition on the intestinal microbial characteristics and the relationship between excretion parameters and microbial characteristics during late pregnancy, the diversity of the microbial communities in the feces was evaluated using 16S rRNA gene sequencing. After quality control of the fecal 16S rRNA gene sequencing, a total of 1,264,439 effective tags were obtained from 28 fecal samples and each sample was covered by an average of 45,160 effective tags. The coverage values of all samples averaged 99.99%, so the sequencing results fully reflect the distribution of fecal bacteria. The α-diversity indexes, including those depicted in Figure 3, indicate there was no significant difference among the four groups in Shannon (*p* = 0.28) and Chao (*p* = 0.21). This means there were no differences in the microbial diversity and abundance, and these results were also approved by Simpson and Ace, respectively.

**Figure 4:**
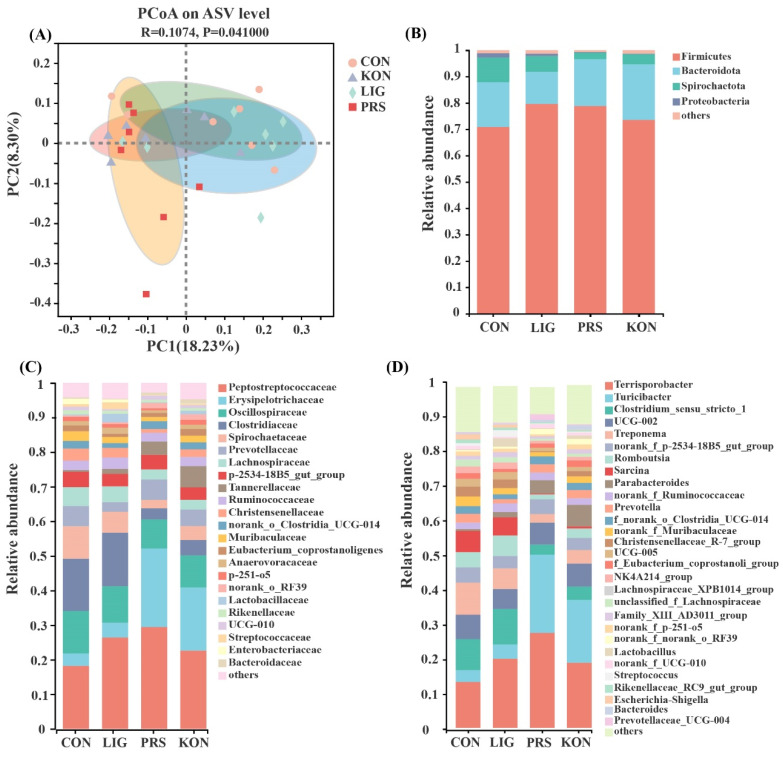
Comparison of the composition of the fecal microbiome (*n* = 7). The principal coordinate analysis (PCoA) plots of the four groups (**A**). Microbial structure at the phylum level (**B**), family level (**C**), and genus level (**D**) in the feces of sows. CON, control; LIG, lignocellulose; PRS, resistant starch; KON, konjaku flour.

**Figure 5:**
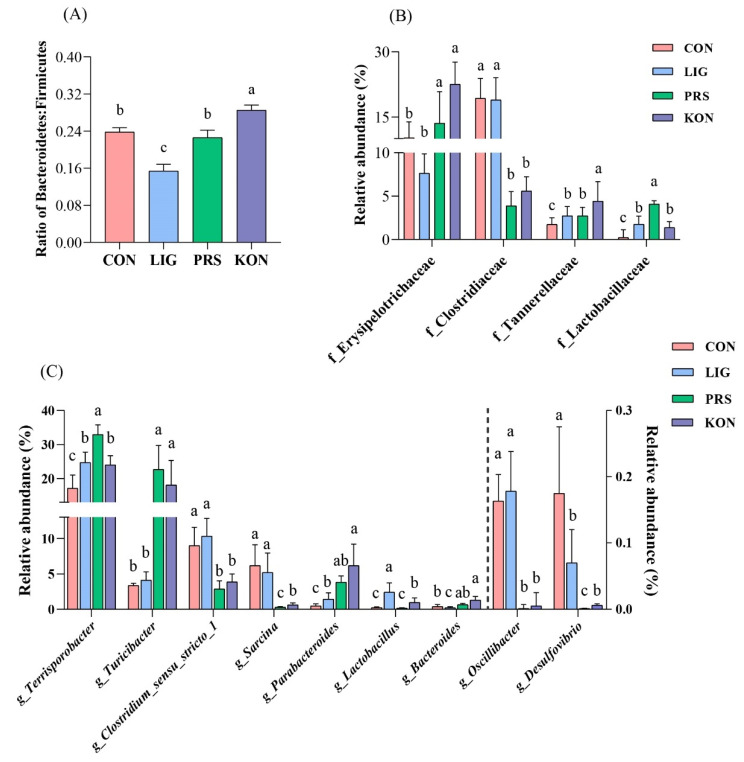
Differential microbial analysis of the feces of late pregnant sows (*n* = 7). The ratio of Bacteroidetes to Firmicutes (**A**), and differential microbial analysis at the family level (**B**) and genus level (**C**) between CON and PRS. CON, control; LIG, lignocellulose; PRS, resistant starch; KON, konjaku flour. Differences in superscript letters for the peer data indicate that the difference is significant (*p* < 0.05).

Next, principal coordinate analysis (PCoA) of the unweighted UniFrac distances with the Adonis test was performed on the ASV abundance, revealing that there was a clear separation of the microbial community among the four treatments (Figure 4A). Firmicutes and Bacteroidetes were the most dominant phyla in the fecal samples, followed by Spirochaetes and Proteobacteria (Figure 4B). At the family level, the dominant families were Peptostreptococcaceae, Erysipelotrichaceae, Oscillospiraceae, and Clostridiaceae, accounting for more than 65% of the total abundance (Figure 4C). The top five genera across the four groups were *Terrisporobacter*, *Turicibacter*, *Clostridium_sensu_stricto_1*, *UCG-002*, and *Treponema* (Figure 4D). Figure 5A shows that the ratio of Bacteroidetes to Firmicutes in KON (0.29) was higher than those of CON (0.23) and LIG (0.15) (*p* < 0.05). 

Given that the three dietary supplementations relieved constipation, a Wilcoxon rank-sum test analysis of the differentially abundant bacteria at the family and genus level was conducted among the four groups. At the family level (Figure 5B), the relative abundances of Erysipelotrichaceae and Tannerellaceae were higher in PRS and KON while the families Clostridiaceae and Oscillospiraceae tended to be decreased compared with CON. The relative abundance of Lactobacillaceae also tended to be increased in PRS and LIG compared to CON. At the genus level (Figure 5C), the relative abundance of *Clostridium_sensu_stricto_1*, *Sarcina*, *Desulfovibrio*, and *Oscillibacter* tended to be reduced in PRS and KON compared with CON. Conversely, PRS and KON had a higher abundance of *Terrisporobacter*, *Parabacteroides*, and *Bacteroides* compared with CON. Moreover, the abundance of *Lactobacillus* also tended to be increased in KON and LIG compared to CON. Interestingly, the relative abundance of *Turicibacter* was significantly higher in PRS and KON compared with CON and LIG. Moreover, PRS presents the highest relative abundance of *Turicibacter* among the four groups (*p* < 0.01).

### 3.5. Effects of Different Fiber Sources on the Fecal SCFAs Profiles of Sows

**Figure 6:**
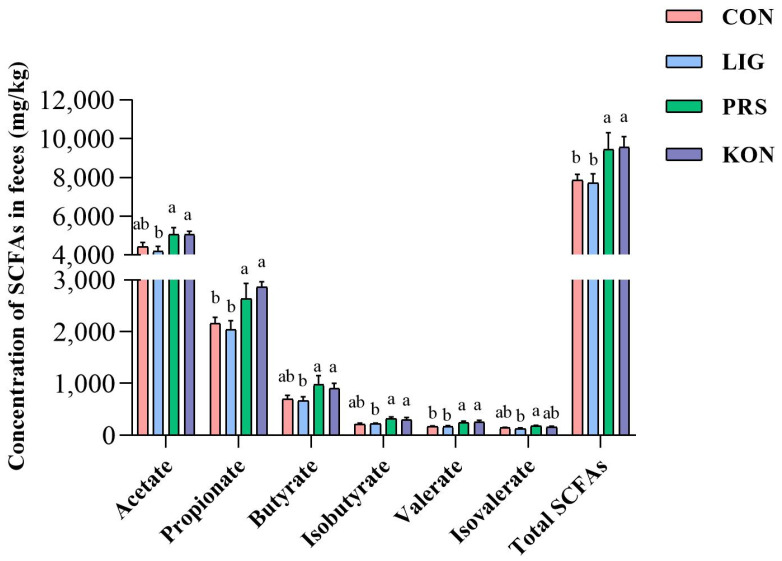
Characteristics of the short-chain fatty acids (SCFAs) profile in the feces of sows after dietary fiber treatment (*n* = 7). CON, control; LIG, lignocellulose; PRS, resistant starch; KON, konjaku flour. Differences in superscript letters for the peer data indicate that the difference is significant (*p* < 0.05).

Bacterial fermentation of dietary fiber in the hindgut results in the production of SCFAs such as acetate, propionate, butyrate, and valerate. As shown in Figure 6, the concentrations of acetate, propionate, butyrate, isobutyrate, valerate, and total SCFAs in PRS and KON were higher than that in LIG and CON. As an example, the total SCFAs in PRS and KON (9364.01 and 9629.4883 mg/kg) were higher than that in CON and LIG (7844.03 and 7714.50 mg/kg) (*p* < 0.05). Moreover, PRS had the highest level of butyrate and isobutyrate among the four groups.

### 3.6. Effects of Different Source Fibers on the Serum Inflammation Factors and Endotoxin of Sows

The concentrations of IL-6, IL-10, TNF-α, and endotoxin in serum are presented in Table 3. There was no significant difference in serum IL-6. CON had the highest concentration of TNF-α in serum. Moreover, the concentration of TNF-α presented the lowest level while IL-10 presented the highest level in PRS. There was no significant difference in the concentration of endotoxin between PRS and KON, but their levels were lower than that of LIG and CON.

### 3.7. Correlation between Defecation Performances, Gut Regulatory Factors, Inflammation Factors, and Differential Bacteria

**Figure 7:**
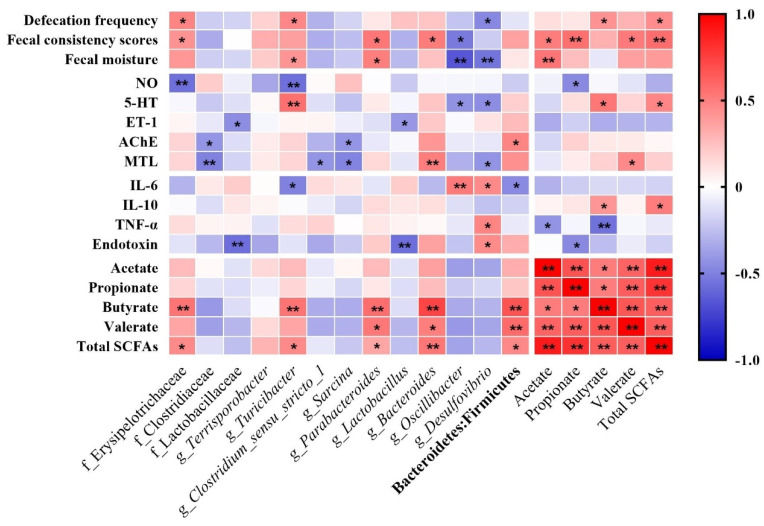
Correlation between fecal microbiota and defecation performances and serum parameters in Spearman correlation analysis. 5-HT, 5-hydroxytryptamine; NO, nitrogen monoxide; ET-1, endothelin-1; AChE, acetylcholinesterase; MTL, motilin; IL-6, interleukin-6; L-10, interleukin-10; TNF-α, tumor necrosis factor α. Red indicates a positive correlation; blue indicates a negative correlation. *, 0.01 < *p* ≤ 0.05; **, 0.001 < *p* ≤ 0.01.

Figure 7 presents the Spearman correlation analyses of the defecation performances, gut regulatory factors, main differential bacteria, and different kinds of and total SCFAs. Defecation frequency was positively correlated with the relative abundance of Erysipelotrichaceae and *Turicibacter* but negatively correlated with that of *Desulfovibrio*. There were statistically significant correlations between the fecal consistency scores and Erysipelotrichaceae (*p* < 0.05), *Bacteroides* (*p* < 0.05), and *Parabacteroides* (*p* < 0.05). Fecal moisture was increased by the relative abundance of *Turicibacter* (*p* < 0.05) and *Parabacteroides* (*p* < 0.05) but decreased by that of *Oscillibacter* (*p* < 0.01) and *Desulfovibrio* (*p* < 0.01). Regarding gut neurotransmitters, the concentration of 5-HT in serum was positively correlated with the abundance of *Turicibacter* but negatively related to that of *Oscillibacter* (*p* < 0.05) and *Desulfovibrio* (*p* < 0.05). The concentration of NO was negatively correlated with Erysipelotrichaceae (*p* < 0.01) and *Turicibacter* (*p* < 0.01). Regarding the gastrointestinal regulatory peptides, the concentration of MTL was positively correlated with Bacteroides (*p* < 0.05) but negatively correlated with Clostridiaceae (*p* < 0.01). The AchE was positively correlated to the ratio of Bacteroidetes: Firmicutes (*p* < 0.05). In terms of the serum inflammation factors and endotoxin, the concentration of IL-6 was increased by the relative abundances of *Oscillibacter* (*p* < 0.01) and *Desulfovibrio* (*p* < 0.05) while it was decreased by *Turicibacter* (*p* < 0.05) and the ratio of Bacteroidetes:Firmicutes (*p* < 0.05). Moreover, the concentration of TNF-α in serum was positively correlated with the abundance of *Desulfovibrio* (*p* < 0.05). Moreover, the relative abundances of *Lactobacillus* and Lactobacillaceae significantly decreased the serum endotoxin (both *p* < 0.01). 

The concentration of SCFAs also showed a strong connection with intestinal microbiota in the correlation analysis. Valerate was positively connected with *Parabacteroides* (*p* <0.05), *Bacteroides* (*p* < 0.05), and the ratio of Bacteroidetes:Firmicutes (*p* < 0.01). Moreover, butyrate was positively promoted by Erysipelotrichaceae, *Turicibacter, Parabacteroides, Bacteroides*, and the ratio of Bacteroidetes:Firmicutes (*p* < 0.01). Total SCFAs was also positively influenced by these bacteria groups (*p* < 0.05).

In addition, the correlation analyses of the fecal SCFAs and defecation performance and serum parameters revealed that the fecal scores were positively correlated with acetate, propionate, valerate, and total SCFAs. Fecal moisture was strongly promoted by acetate. The defecation frequency also had a significant positive relation with butyrate and total SCFAs. Moreover, serum 5-HT was positively correlated with fecal butyrate and total SCFAs. Serum MTL was positively affected by valerate while serum NO was negatively influenced by propionate. As for the levels of pro- and anti-inflammation factors in serum, the concentration of IL-10 was strongly induced by butyrate and total SCFAs while the concentration of TNF-α was negatively influenced by acetate and butyrate. The concentration of serum endotoxin was also negatively correlated with propionate in the feces. 

## 4. Discussion

Constipation during late pregnancy has a series of negative consequences on both the mother and offspring [1,7,26]. Dietary fiber is regarded as an effective nutrient in the maternal diet as it not only promotes fecal moisture in late gestating sows but also alleviates pathological constipation in the rat model [17,27]. However, few studies have focused on the effects of dietary fiber on the constipation of late pregnant sows. In the present study, diets that included three different fiber sources (LIG, PRS, and KON) were fed to sows in the last 4 weeks of gestation. Our study revealed that the different fiber sources alleviated constipation to a different extent. PRS was found to be the best among the three different fibers at promoting the defecation frequency, improving the fecal consistency, and reducing the inflammation of sows. Additionally, consistent with the results of in vitro fermentation [18], the different sources of fiber specifically changed the fecal microbiota of sows. To our knowledge, this is the first study to investigate such effects.

During pregnancy, gut motility is inhibited due to enlargement of the uterus and its contents exerting pressure on the abdominal and back muscles, causing a prolonged GI transit time [7]. A prolonged GI transit time is also accompanied by increased water absorption, low defecation frequency, and hard stools [28]. In this study, the defecation frequency of sows was increased by supplementing LIG, PRS, or KON in the diet, which is consistent with previous studies in which higher-fiber diets had a positive effect on the GI transit time of constipated rats [29]. In recent studies, PRS and KON have been shown to effectively relieve clinical symptoms in patients with constipation and promote gut motility in carbon-induced and loperamide-induced rat models of constipation [27,30]. The dietary LIG, PRS, and KON supplementation also affected the release of gut regulatory factors. The concentrations of 5-HT and MTL in serum were positively related to the defecation frequency of sows in our study. 5-HT is an important paracrine messenger and neurotransmitter involved in the modulation of GI motility and enteric secretory reflexes, and more than 90% of the body’s 5-HT is synthesized in the gut [31,32]. MTL is also involved in GI secretion and motility [33]. Therefore, our findings support the hypothesis that the intake of dietary fiber in late gestation in sows stimulated the release of gut motility regulatory factors, increased the fecal moisture content, and increased the defecation frequency.

Recent studies have shown a strong connection between constipation and intestinal microorganisms [11,29]. Stool consistency and defecation frequency were associated with gut microbial richness in women, and the related bacterial groups were involved in colonic fermentation [10,24]. Moreover, the colonic transit time has been related to bacterial metabolism and mucosal turnover in the gut [15]. Therefore, it is speculated that different fibers affect gut motility by differently affecting the gut microbiome. A decreased ratio of Bacteroidetes to Firmicutes was found during constipation in the gut microbial structure in humans [13,34], which is consistent with the present study showing that dietary fiber KON increased the ratio of Bacteroidetes to Firmicutes. Moreover, resistant starch from bananas could improve the diversity of intestinal microbiota and upregulate the Bacteroides/Firmicutes ratio [35]. In our study, maternal PRS and KON supplementation increased the relative abundance of *Prevotellaceae*, *Turicibacter*, *Bacteroides*, *Parabacteroides*, and *Lactobacillus* and decreased the level of *Clostridium_sensu_stricto_1*, *Desulfovibrio*, and *Oscillibacter*. These changes in the microbiome were related to changes in the gut motility factors. In our study, the levels of MTL and 5-HT were found to positively correlate with the ratio of Firmicutes to Bacteroidetes and *Prevotellaceae*, *Turicibacter*, *Parabacteroides*, and *Bacteroides*. Yano et al. found that approximately 50% of gut-derived 5-HT was regulated by the gut microbiota, particularly spore-forming bacteria dominated by the families Clostridiaceae and Turicibacteraceae, with downstream consequences for host intestinal motility [36]. Moreover, *Turicibacter* has been reported as a gut bacterium that expresses a neurotransmitter sodium symporter-related protein with sequence and structural homology to mammalians, which promoted host 5-HT biosynthesis [37]. *Parabacteroides* and *Bacteroides* were regarded as butyrate-producing bacteria [38,39], and the number of *Bacteroides* was also significantly less abundant in feces from patients with functional constipation compared with healthy controls [40,41]. In our study, PRS and KON sows had lower serum endotoxin levels. This corresponds with the low abundance of *Desulfovibrio* in PRS and KON. *Desulfovibrio* has been identified as an important endotoxin producer [42], and its abundance is highly linked to the development of constipation both in animal and human models [43,44]. Moreover, the higher levels of beneficial bacteria in the intestine might modulate the total microbial composition, suppressing the proliferation of opportunistic pathogens, and inhibiting toxicity production. Another potential mechanism for the reduction of harmful bacteria is the increased production of SCFAs after fiber treatment, which possess antimicrobial activity [45]. SCFAs also have the potential to promote the intestinal peristaltic contraction frequency and relieve constipation in animals [27]. Therefore, our study demonstrates that maternal dietary fiber during late pregnancy promoted the proliferation of specific microflora, which then promoted the release of specific gut regulatory factors in the gut, which in turn promoted intestinal peristalsis. PRS is the best fiber among the three different sources of fiber at relieving the constipation of sows.

The changes in the gut microbiome among the different fiber treatments during late gestation may be ascribed to the molecular structure of the different fibers [46]. Hamaker and colleagues proposed that some unique chemical structures within fiber molecules match the gene clusters of specific microbial species, which can predict changes in the microbiota composition [47]. Therefore, the microbial changes caused by different fibers in this study may be related to the molecule groups of fibers. KON is a kind of polysaccharide composed of glucose and mannose and bound by a p-1, 4-glycosidic, which is easily degraded by (1,4)-β-D-mannanase [16]. Our study showed that the relative abundances of *Lactobacillus* and *Bacteroides* were higher in KON compared with CON. KON could easily be fermented by analogous types of microbiota, which produce (1,4)- β-D-mannanase or cellulase [48]. Moreover, β-glucan can contribute to the growth of *Bacteroides* and *Lactobacillus*, and this effect is connected with the molecular weight of β-glucan [49]. *Bacteroides* have been considered as a candidate for next-generation probiotics for health in humans [50]. PRS is a type 3 resistant starch obtained by gelatinization and recrystallization of amylose and amylopectin. The properties of this type are directly related to the starch characteristics, such as the amylose content and the degree of polymerization. Moreover, type 3 resistant starch is particularly relevant to the retrograded amylose since linear chains that form double-helical structures can align after gelatinization, leading to the inaccessibility of the glycosidic linkages to digestive enzymes [51]. The fermentation of resistant starch in the human large intestine depends on the presence of amylolytic bacteria. *Bifidobacterium adolescentis* and *Ruminococcus bromii* have shown the ability to use resistant starch [52], and the microbial community that degrades the type 3 resistant starch was also composed of highly amylolytic microbes (*Prevotella*, *Bacteroides*, and *Lactobacillus*) [53]. In this study, the relative abundance of *Bacteroides* and *Parabacteroides* increased after PRS treatment, which is consistent with a previous report [54]. *Parabacteroides*, belonging to Bacteroidetes, is a saccharolytic anaerobe that produces acetate, butyrate, and succinate as end products of carbohydrate fermentation [38]. Another interesting result is that the relative abundance of *Turicibacter* in PRS was the highest among the four groups. As we discussed above, *Turicibacter* has been reported as an important genus in health regulation through promotion of host 5-HT biosynthesis. Moreover, *Turicibacter* was significantly increased after resistant starch treatment in pig and rat models, which may be the target microorganism of resistant starch [35,55,56]. Therefore, we speculate that *Turicibacter* is the key bacterial community of PRS-mediated relief of constipation in sows. The higher relative abundance of *Bacillus* in LIG might be associated with the high cellulose contents in LIG, which are slowly fermented by gut bacteria and stimulate the growth of fiber-degraded bacteria [18]. 

Changes in intestinal motility might also be mediated by microbial-derived metabolites [44]. These SCFAs include acetate, propionate, and butyrate, and have been demonstrated to be key regulators of host metabolism and immunity by regulating the release of gastrointestinal hormones [39,57,58]. Moreover, SCFAs are also an energy source for the host, with various effects on myenteric neurons and motility [57]. The current study demonstrated that the feces of sows fed PRS and KON tended to have greater propionate, butyrate, and total SCFAs levels, which might explain the higher defecation frequency and higher serum level of 5-HT and MTL than CON [12,59]. About 90% of 5-HT in the human body is produced by enterochromaffin cells (ECs) of the gut [31], and SCFAs can promote the release of 5-HT from EC cells by stimulating 5-HT3 receptors located on the vagal sensory fibers [60]. Among these kinds of SCFAs, butyrate increases propagating contractions in the proximal colon of rats and has been used to treat various gastrointestinal motility disorders [57]. In this study, the concentration of butyrate was highest in the feces of PRS. Consistently, PRS had the highest level of 5-HT in serum and the highest defecation frequency among the four groups. Additionally, the correlation analyses showed a strong connection between butyrate and serum 5-HT and the defecation frequency. Thus, a potential mechanism for constipation relief during late pregnancy by PRS and KON is increased 5-HT release from gut cells induced by microbial SCFAs, especially butyrate [44,60]. The concentration of fecal SCFAs in KON and PRS was higher than that in LIG, which can be explained by the different ratios of soluble fiber/insoluble fiber between KON, PRS, and LIG, because LIG contains higher levels of insoluble fiber cellulose [18]. There was no difference in the SCFAs production between PRS and KON, and the total SCFA of KON was numerically higher, which is consistent with a previous report [54]. Conversely, the defecation frequency and serum gut motility regulator in PRS are higher than that in KON. In previous studies, PRS was continuously broken down to release SCFAs, and the fermentation curve was prolonged compared with KON [18,22]. Moreover, PRS tends to produce more butyrate in microbial fermentation, which is more effective at promoting 5-HT production and gut motility [44]. Therefore, PRS is better than KON at promoting intestinal peristalsis and relieving constipation, due to the constant stimulation of stable and continuous SCFAs [22], and a higher level of butyrate. At the same time, resistant starch with a high amylose level can also increase intestinal SCFAs absorption [61], which can also contribute to the higher efficiency of PRS in promoting intestinal motility. Moreover, the continuous release of SCFAs also continuously promotes the growth of beneficial bacteria and depresses the proliferation of opportunistic pathogens [52]. 

Microbiota-derived SCFAs have been shown to reduce the inflammatory response by inhibiting pro-inflammatory factors, such as TNF-α, IL-1β, and IL-6, and maintain intestinal homeostasis by protecting the integrity of the epithelial barrier [62,63]. A previous study showed that maternal consumption of dietary fiber significantly reduced markers of intestinal permeability (endotoxin) in sow serum, and systemic inflammation (IL-6 and TNF-α) in sow feces and serum [64]. In our study, PRS treatment had a lower inflammation condition, reflected by the lower serum TNF-α and the highest serum IL-10. IL-10 is an anti-inflammation factor produced by regulatory T lymphocytes, monocytes, and macrophages, which mainly inhibits the production of inflammatory cytokines, such as TNF-α [45]. Moreover, a strong positive connection between serum IL-10 and fecal butyrate was observed in this study, which is consistent with the results showing that microbiota-derived butyrate promotes microbiota antigen-specific Th1 cell IL-10 production, mediated by G-protein-coupled receptors [65].

## 5. Conclusions

Dietary fiber sources have different effects on the constipation of sows during late gestation. The relieving effect of PRS and KON on constipation was better than LIG and CON. PRS and KON increased the relative abundances of *Bacteroides*, *Parabacteroides*, and *Turicibacter*, and decreased the endotoxin-producing bacteria *Desulfovibrio* and *Oscillibacter*. PRS and KON also produced higher levels of butyrate and total SCFAs, and promoted the release of 5-HT, ET-1, and MTL, which stimulated the gut motility of sows. Moreover, PRS and KON reduced the inflammation response by inhibiting the production of pro-inflammation factors. Our findings may provide a theoretical reference and guide for the use of specific fiber sources in the maternal diet to improve the gut movements of the late-gestating mother.

## Figures and Tables

**Table 1:** Ingredients and nutrient composition of the experimental diets (%, as-fed basis).

Items	CON ^1^	LIG ^1^	PRS ^1^	KON ^1^
Ingredient (%)				
Corn	53.50	53.50	53.50	53.50
Soybean meal	13.00	13.50	13.00	13.00
Soybean oil	2.00	2.00	2.00	2.00
Wheat bran	28.00	26.00	26.00	26.00
Lignocellulose		1.50		
Resistant starch			2.00	
Konjaku flour				2.00
Limestone	1.25	1.25	1.25	1.25
Dicalcium phosphate	1.10	1.10	1.10	1.10
NaCl	0.50	0.50	0.50	0.50
L-Lysine HCl	0.05	0.05	0.05	0.05
Methionine	0.04	0.04	0.04	0.04
Threonine	0.05	0.05	0.05	0.05
Tryptophan	0.01	0.01	0.01	0.01
Premix ^2^	0.50	0.50	0.50	0.50
Total (%)	100	100	100	100
Nutrient composition				
DE ^3^ (Mcal/kg)	3.29	3.25	3.24	3.24
CP ^3^ (%)	15.50	15.38	15.29	15.33
Ca ^3^ (%)	0.78	0.78	0.78	0.78
P ^3^ (%)	0.75	0.73	0.73	0.73
Lys ^3^ (%)	0.73	0.73	0.72	0.72
Met ^3^ (%)	0.30	0.30	0.29	0.29
Thr ^3^ (%)	0.58	0.58	0.57	0.57
Trp ^3^ (%)	0.17	0.17	0.17	0.17
NDF ^3^ (%)	15.64	16.23	16.12	16.48
ADF ^3^ (%)	4.92	5.76	4.77	4.93
TDF ^3^ (%)	17.89	18.86	18.69	18.72

^1^ CON, control; LIG, lignocellulose; PRS, resistant starch; KON, konjaku flour. ^2^ Premix provided per kilogram of diet: vitamin A, 11 000 IU; vitamin D3, 1500 IU; vitamin E, 15 IU; vitamin K3, 1.6 mg; vitamin B1, 1.5 mg; vitamin B2, 3.0 mg; vitamin B6, 1.5 mg; vitamin B12, 0.015 mg; niacin, 22.5 mg; D-pantothenic acid, 15 mg; folic acid, 2.5 mg; biotin, 0.2 mg; Fe, 85 mg; Cu, 7.5 mg; Zn, 75 mg; Mn, 35 mg; I, 0.5 mg; Se, 0.3 mg. ^3^ DE: digestion energy, CP: crude protein, Ca: calcium, P: phosphorus, Lys: lysine, Met: methionine, Cys: cysteine, Thr: threonine, Trp: tryptophan, NDF: neutral detergent fiber, ADF: acid detergent fiber, TDF: total dietary fiber.

**Table 2:** Effects of different fiber sources on the reproductive performance and parturition duration of sows (*n* = 20).

Items	CON ^1^	LIG ^1^	PRS ^1^	KON ^1^	SEM	*p*-Value
Total piglets (*n*)	15.78	14.78	15.11	14.33	0.33	0.46
Born alive (*n*)	14.26	13.78	14.11	13.88	0.32	0.96
Stillborn number (*n*)	1.81	1.00	0.61	0.68	0.14	0.08
Stillborn rate (%)	11.29 ^a^	8.47 ^ab^	3.94 ^b^	4.49 ^b^	0.94	0.02
Litter weight (kg)	19.87	19.38	19.26	19.07	0.36	0.88
Average bodyweight (kg)	1.40	1.45	1.39	1.46	0.02	0.62
Duration of parturition (min)	397.69 ^b^	399.07 ^b^	286.47 ^a^	282.85 ^a^	19.35	0.03

^1^ CON, control; LIG, lignocellulose; PRS, resistant starch; KON, konjaku flour. ^a,b^ Mean values within a row with different letters differ at *p* < 0.05. SEM, standard error of the mean.

**Table 3:** Effects of different fiber sources on the serum inflammation factors and endotoxin of late pregnant sows (*n* = 7).

Item	CON ^1^	LIG ^1^	PRS ^1^	KON ^1^	SEM	*p*-Value
IL-6 (pg/mL) ^2^	12.71	12.10	11.32	10.77	1.32	0.11
IL-10 (pg/mL) ^2^	18.55 ^c^	19.81 ^b^	23.55 ^a^	21.06 ^b^	1.05	0.04
TNF-α (pg/mL) ^2^	132.11^a^	115.33 ^b^	109.52 ^c^	115.34 ^b^	6.88	0.01
Endotoxin (EU/L) ^2^	36.42 ^a^	34.31 ^b^	31.03 ^c^	31.71 ^c^	1.26	0.03

^1^ CON, control; LIG, lignocellulose; PRS, resistant starch; KON, konjaku flour. ^2^ IL-6, interleukin-6; IL-10, interleukin-10; TNF-α, tumor necrosis factor-α. ^a–c^ Mean values within a row with different letters differ at *p* < 0.05. EUs, endotoxin units; SEM, standard error of the mean.

## Data Availability

The data presented in this study are available on request from the corresponding author.

## Abbreviations

LIG, Lignocellulose; PRS, Resistant starch; KON, Konjaku flour; SCFAs, Short-chain fatty acids; NO, Nitrogen monoxide; ET-1, Endothelin-1; 5-HT, 5-Hydroxytryptamine; AChE, Acetylcholinesterase; MTL, Motilin; ASV, Amplicon sequence variant; BSS, Bristol stool scale; EC, Enterochromaffin cells; IL-6, Interleukin-6; IL-10, Interleukin-10; Interleukin-1β, IL-1β; TNF-α, Tumor necrosis factor-α.
